# Wie die Umsetzung einer Schule für Menschen mit Parkinson-Krankheit gelingen kann – Ergebnisse eines Konsensusverfahrens und einer formativen Evaluation

**DOI:** 10.1007/s00115-024-01639-z

**Published:** 2024-03-14

**Authors:** Tanita Gerschel, Scally Prokop, Lara Schulze, Tim Feige, Anja Zschieschang, Michael Thomas Barbe, Robert Bitterlich, Julian Caffier, Ilona Csoti, Carsten Eggers, Heiko Gaßner, Eileen Gülke, Tom Hähnel, Heinz Herbst, Angela Jochim, Anni Kiparski, Martin Klietz, Alexa von Liel, Paul Lingor, Kai Loewenbrück, Walter Maetzler, Dominik Pürner, Christoph van Riesen, Henning Schmitz-Pfeiffer, Thorsten Süß, Lars Tönges, Daniel Weiß, Martin Wolz, Björn Falkenburger

**Affiliations:** 1 Klinik und Poliklinik für Neurologie, Universitätsklinikum Carl Gustav Carus an der Technischen Universität Dresden, Dresden, Deutschland; 2 Deutsches Zentrum für Neurodegenerative Erkrankungen (DZNE), Standort Dresden, Dresden, Deutschland; 3 Klinik und Poliklinik für Kinderchirurgie, Universitätsklinikum Carl Gustav Carus an der Technischen Universität Dresden, Dresden, Deutschland; 4 Klinik für Neurologie, Universität zu Köln, Medizinische Fakultät und Uniklinik Köln, Köln, Deutschland; 5 Universitätsklinikum Hamburg Eppendorf, Hamburg, Deutschland; 6 Fachabteilung für Neurologie, Fachklinik für Parkinson, Gertrudis Klinik, Biskirchen, Deutschland; 7 Klinik für Neurologie, Knappschaftskrankenhaus Bottrop, Bottrop, Deutschland; 8 Molekulare Neurologie, Arbeitsgruppe Bewegungsanalyse und digitale Medizin, Universitätsklinikum Erlangen, Erlangen, Deutschland; 9 Fraunhofer Institut für integrierte Schaltungen IIS, Fraunhofer IIS, Erlangen, Deutschland; 10 Klinik und Poliklinik für Neurologie, Universitätsklinikum Hamburg Eppendorf, Hamburg, Deutschland; 11 Neurozentrum Stuttgart, Stuttgart, Deutschland; 12 Klinik und Poliklinik für Neurologie, Klinikum rechts der Isar der Technischen Universität München, München, Deutschland; 13 Klinik für Neurologie mit Klinischer Neurophysiologie, Medizinische Hochschule Hannover, Hannover, Deutschland; 14 Klinik für Neurologie, Universitätsklinikum Schleswig-Holstein Campus Kiel und Christian-Albrechts-Universität Kiel, Kiel, Deutschland; 15 Klinik für Neurologie, Universitätsklinikum Göttingen, Göttingen, Deutschland; 16 Neurologisches Fachkrankenhaus für Bewegungsstörungen/Parkinson, Beelitz, Deutschland; 17 Klinik für Neurologie, St. Josef-Hospital, Ruhr-Universität Bochum, Bochum, Deutschland; 18 Zentrum für Proteindiagnostik (ProDi), Ruhr-Universität Bochum, Bochum, Deutschland; 19 Klinik für Neurologie, Universitätsklinikum Tübingen, Tübingen, Deutschland; 20 Klinik für Neurologie und Geriatrie, Elblandklinikum Meißen, Meißen, Deutschland

**Keywords:** Patientenbeteiligung, Chronische Erkrankungen, Expertenpanel, Qualitätsmanagement, Selbstmanagement, Patient participation, Chronic disease, Expert panel, Quality management, Self-management education

## Abstract

**Hintergrund:**

Die Parkinson-Krankheit ist als weltweit am schnellsten zunehmende neurodegenerative Erkrankung gesellschaftlich hoch relevant. Für eine erfolgreiche Behandlung ist die aktive Beteiligung der Patient*innen erforderlich. Patientenschulen werden bei vielen chronischen Erkrankungen wie Diabetes erfolgreich eingesetzt und könnten auch Menschen mit der Parkinson-Krankheit Fähigkeiten vermitteln, besser mit der Krankheit umzugehen und an Therapieentscheidungen teilzunehmen.

**Material und Methoden:**

Um die Implementierung eines Konzepts für eine Patientenschule für Menschen mit der Parkinson-Krankheit vorzubereiten, wurde ein strukturiertes Konsensusverfahren durchgeführt und ein Pilotprojekt formativ evaluiert. Das strukturierte Konsensusverfahren mit deutschlandweit rekrutierten Expert*innen gliederte sich in eine 1. und 2. Onlinebefragung sowie eine abschließende Konsensuskonferenz. Die formative Evaluation erfolgte durch drei Fokusgruppen. Die Transkripte dieser Gruppeninterviews wurden computergestützt mittels inhaltlich-strukturierender qualitativer Inhaltsanalyse ausgewertet.

**Ergebnisse:**

In dem Konsensusverfahren konnten 59 Aussagen konsentiert werden, insbesondere zu den Inhalten einer Patientenschule und zu einer Gruppengröße von 6 bis 8 Personen. Lediglich zwei Aussagen wurden nicht konsentiert. Aus der formativen Evaluation waren eine tendenziell positive Haltung gegenüber einem digitalen Schulungsformat und eine sehr positive Bewertung der Inhalte ableitbar.

**Diskussion:**

Insgesamt konnten wichtige Empfehlungen für eine Patientenschule formuliert werden. Zu den Themen Format, Einschlusskriterien, Gruppenzusammensetzung und Einbeziehung von Angehörigen ist dagegen eine weitere Betrachtung erforderlich.

**Zusatzmaterial online:**

Die Online-Version dieses Beitrags (10.1007/s00115-024-01639-z) enthält zusätzliches Material. Beitrag und Zusatzmaterial stehen Ihnen auf www.springermedizin.de zur Verfügung. Bitte geben Sie dort den Beitragstitel in die Suche ein, das Zusatzmaterial finden Sie beim Beitrag unter „Ergänzende Inhalte“.

## Abstract (en)

**Background:**

As the most rapidly increasing neurodegenerative disease worldwide, Parkinson’s disease is highly relevant to society. Successful treatment requires active patient participation. Patient education has been successfully implemented for many chronic diseases, such as diabetes and could also provide people with Parkinson’s disease with skills to manage the disease better and to participate in shared decision making.

**Material and methods:**

To prepare the implementation of a concept for patient education for people with Parkinson’s disease, a structured consensus study was conducted and a pilot project formatively evaluated. The structured consensus study included experts from all over Germany. It consisted of two online surveys and an online consensus conference. The formative evaluation was conducted as three focus groups. Transcripts were evaluated using content-structuring qualitative content analysis.

**Results:**

From the consensus procedure 59 consented statements emerged, mainly regarding the contents of a patient school and a group size of 6–8 persons. Only two statements could not be consented. The formative evaluation detected a tendency towards a positive attitude for a digital training format and a very positive evaluation of the contents.

**Discussion:**

Overall, important recommendations for a patient school can be drawn from this study. The following subjects require further investigation: format, inclusion criteria, group composition and inclusion of caregivers.

Die Parkinson-Krankheit ist weltweit die am schnellsten zunehmende neurodegenerative Erkrankung [1]; die Symptome können zu einer hohen Beeinträchtigung der Patient*innen im alltäglichen Leben führen [2, 3]. Die gezielte Kompetenzförderung kann den Umgang mit der Erkrankung erleichtern. Hierzu wird für die Diabetes-Erkrankung seit über 80 Jahren eine Patientenschule genutzt [4]. Auch eine Schule für Menschen mit der Parkinson-Krankheit könnte die Lebensqualität der Patient*innen verbessern [5], jedoch existiert diesbezüglich bislang in Deutschland kein eindeutiges Konzept [6].

## Hintergrund und Fragestellung

Eine Patientenschule erleichtert durch das Vermitteln notwendiger Fähigkeiten und Fertigkeiten das alltägliche Leben mit der Erkrankung. Dies stärkt die aktive Beteiligung der Patient*innen an Therapieentscheidungen, welche zuletzt notwendig für eine gute Qualität der Patientenversorgung ist [7]. Als Folge dessen kann das Gesundheitssystem von verringerter Inanspruchnahme profitieren [8]. Vor allem jedoch profitieren die Patient*innen selbst von einem solchen Schulungsprogramm; eine Reihe internationaler Studien zeigte eine positive Wirksamkeit auf die Lebensqualität chronisch Erkrankter [5]. Ähnliche Effekte konnten auch für Parkinson-spezifische Schulen nachgewiesen werden [9]. Vor dem Hintergrund der positiven Wirkung förderte die europäische Union von 2003 bis 2005 das Programm „Patient Education for People with Parkinson’s Disease and their Carer“ (PEPP; [10]). Dieses Programm stellte die „self-management education“ (SME) in den Vordergrund und unterstützte Patient*innen dabei, alltagsnotwendige Fähigkeiten zu erlernen. Das Programm wurde nach dem Jahr 2005 nicht dauerhaft in den klinischen Alltag implementiert, bildete in Schweden jedoch die Grundlage für die National Parkinson School. Diese ist bislang das einzige Beispiel für ein landesweit umgesetztes SME-Programm [6].

Für die deutschlandweite Implementierung einer Patientenschule können die bisherigen Programme als Grundlage verstanden werden, jedoch nicht als vollständig übertragbares Konzept. Um Rahmenbedingungen und Anforderungen an eine Schule für Menschen mit der Parkinson-Krankheit sowie mögliche Inhalte einer solchen Schule zu erheben, wurde ein strukturiertes Konsensusverfahren mit Expert*innen der Parkinson-Krankheit durchgeführt. Zur weitergehenden Betrachtung aus dem Blickwinkel von Patient*innen wurde zudem ein formatives Evaluationsverfahren einer als Pilotprojekt durchgeführten Patientenschule für Menschen mit der Parkinson-Krankheit angewendet. Details können dem Methodenteil im Anhang entnommen werden. Der Vergleich beider Verfahren kann Aufschluss über die praktische Implementierung des von den Expert*innen im Konsensusverfahren erwünschten Verfahrens geben.

## Ergebnisse

### Konsensusverfahren

An der deutschlandweiten Expertenbefragung beteiligten sich 56 Expert*innen, wobei 24 an der finalen Konsensuskonferenz teilnahmen. Die Befragung umfasste 61 Aussagen, von denen 30 in der ersten und neun in der zweiten Runde konsentiert wurden. Nur zwei Aussagen konnten auch in der Konsensuskonferenz nicht konsentiert werden. Besonders hervorzuheben ist die einhellige Befürwortung der Etablierung einer Schule für Parkinson-Betroffene. Uneinigkeit herrschte hinsichtlich des Formats der Schule und einer regelmäßigen Wiederholung. Patient*innen bevorzugten ein gemischtes Online- und Präsenzformat. Empfehlungen aus diesem Prozess sind in Tab. 1 und im Anhang aufgeführt.

**Tab. 1:** Vergleich des strukturierten Konsensusverfahrens mit der evaluierten Schule und den Evaluationsergebnissen

Kategorie	Konsensusverfahren	Formative Evaluation
Rahmenbedingungen und Konzept	Regelmäßige Aktualisierung des Konzepts (100 %) Schriftliches Manual (94,3 %). Spezifische Konzepte und Manuale für verschiedene Stadien (93,8 %)	Manualisiertes Schulungskonzept Gemeinsame Schulung aller Stadien
Format der Schulung	Ambulante Durchführung bevorzugt (69,6 %) Hybrides Format (Online und Präsenz) mehrheitlich bevorzugt (56 %) Anpassung nach Bedürfnissen und Gegebenheiten	Gutes Zurechtkommen mit dem Onlineformat Wunsch nach persönlichem Kontakt Vorschlag virtuelle Umsetzung mit Eröffnungs- und Abschlusstermin in Präsenz
Einschluss von Angehörigen	Teilweise Teilnahme befürwortet (78,3 %)	Deutliche Ablehnung einer gemeinsamen Schulung mit Angehörigen
Zeitlicher Rahmen und Struktur	6–8 wöchentliche Module (85,7 %) 60–90 min pro Modul mit Pause (85 %) Wöchentlicher Rhythmus (87 %)	7 wöchentliche 90-Minuten-Module mit Pause Wöchentlicher Rhythmus und Moduldauer positiv bewertet Bedeutung der Pause hervorgehoben
Modulbestandteile, Inhalte und Materialien	Krankheitsspezifische Inhalte mit Selbstmanagementfokus Interaktive Methoden Patientenzentrierte Materialien	Inhalte aus manualisiertem Konzept mit Praxisübungen und realen Beispielen positiv wahrgenommen Wunsch nach Aufgreifen neuester Forschungsergebnisse
Gruppenstruktur	Optimale Gruppengröße von 6–8 Personen identifiziert	Kritik an zu geringer Personenanzahl in der Gruppe mit 3 Personen Lob der „kleinen“ Gruppengröße (5–7)
Schulungsleitung	Multiprofessionelles Team empfohlen	Durchführung durch zwei psychologische Fachkräfte positiv eingeschätzt
Techniken der Wissensvermittlung	Einsatz interaktiver und praxisnaher Methoden befürwortet	Positive Wahrnehmung des Erfahrungsaustausches Wahrnehmung der Wochenaufgaben als überwiegend sinnvoll und in der Umsetzung von eigenen Ressourcen abhängig

Eine ausführlichere Darstellung findet sich in Tab. 2 im elektronischen Zusatzmaterial

### Formative Evaluation

Die relevantesten formativen Evaluationsergebnisse sind in Tab. 1 aufgeführt. Sie basieren auf der inhaltlich-strukturierenden qualitativen Inhaltsanalyse der Transkripte von 13 Zufriedenheitsinterviews und 3 Fokusgruppen mit insgesamt 12 Teilnehmenden. Die digitale Umsetzung stand mit 209 von insgesamt 554 kodierten Textstellen, davon 52 zu technischen Schwierigkeiten, im Fokus. Besonders hervorzuheben ist, dass sich technische Probleme zu Beginn einer neuen Gruppe häuften und im Verlauf merklich abnahmen. Probleme mit dem Ton waren häufiger als Probleme mit der Videoübertragung und wurden als schwerwiegender wahrgenommen. Auch Vorteile wie Zeitersparnis und das Wegfallen der Anfahrt wurden genannt. Zitat zu technischen Schwierigkeiten:

„Was mich immer ein bisschen stört ist, dass diese Rückkopplungen so groß sind, wenn alle das Mikrofon anmachen“ (Fokusgruppe 2).

Zitat zu Vorteilen der technischen Umsetzung:

„Der Vorteil ist, man muss bei Regen nicht raus. Das ist sehr angenehm“ (Fokusgruppe 3).

Anpassungen wie das Versenden von E‑Mail-Links wurden vorgeschlagen und implementiert. Die große Rolle des Erfahrungsaustausches wurde gelobt und die Inhalte der Schulung wurden sehr positiv bewertet. Zitate:

„Könnte nichts herausgestrichen werden. (…) War so gut, wie es war“ (Fokusgruppe 2).

„Die Inhalte waren sehr gut, die haben mir sehr gut gefallen“ (Fokusgruppe 1).

Gegenüber einer gemeinsamen Schulung mit Angehörigen zeichnete sich eine deutlich abwehrende Haltung ab. Zitat:

„Also ich lebe ja allein und das wäre vielleicht meine Tochter, die sich dafür interessieren würde, aber das möchte (…) ich würde das gar nicht wollen, weil ich habe einen ganz anderen Bezug zu der Krankheit als sie und nein, kann ich mir jetzt nicht vorstellen“ (Fokusgruppe 1).

Eine separate Schulung für Angehörige schien nicht allen Interviewten notwendig, vielen jedoch vorstellbar. Bei individuell unterschiedlicher Nutzung der Lernplattform (von 3‑mal während der gesamten Schule bis 3‑mal pro Woche) ist v. a. erwähnenswert, dass auch Teilnehmende ohne Vorbereitung die Inhalte der Videokonferenzen verständlich fanden. Der wöchentliche Rhythmus der Schulung und die Dauer der Videokonferenzen von 90 min wurden als angemessen beurteilt, wobei die große Bedeutung der Pause betont wurde.

## Diskussion

Der Vergleich der konsentierten Vorstellungen der Expert*innen des Konsensusverfahrens mit dem bereits umgesetzten, evaluierten Schulungsprogramm zeigt eine hohe Übereinstimmung zwischen den im Konsensusverfahren erarbeiteten Vorstellungen und dem evaluierten Schulungsprogramm. Wesentliche Übereinstimmungen sind in Tab. 1 zusammengefasst. Sie betreffen die Nutzung eines Manuals, den modularen Aufbau, die multiprofessionelle Teamzusammensetzung und die optimale Gruppengröße von 6 bis 8 Personen. Die Inhalte wurden sowohl im Konsensusverfahren als auch in der formativen Evaluation positiv bewertet.

### Format

Ein wesentlicher Diskussionspunkt bleibt das Format der Patientenschule. Bisherige Patientenschulen wurden fast immer in einem Präsenzformat durchgeführt [6]. Während reine Online- oder Präsenzformate jeweils Vor- und Nachteile haben, wurde ein gemischtes Modell als Alternative vorgeschlagen. Dies könnte Onlineelemente mit Eröffnungs- und Abschlussveranstaltungen in Präsenz kombinieren.

Dass ein online umgesetztes Konzept sowohl für berufstätige als auch für mobilitätseingeschränkte Patient*innen von Vorteil sein kann, konnte bereits eine Studie von Derollez et al. [11] zeigen. Allerdings wurde der direkte Austausch der Patient*innen bereits innerhalb der ersten Onlinebefragung als wichtiges Element der Patientenschule benannt, und auch andere Patientenschulen beschreiben den persönlichen Austausch als ein wesentliches Erfolgskriterium [12]. Ein ausschließliches Online- oder Präsenzformat scheint daher im Rahmen einer Patientenschule für Menschen mit der Parkinson-Krankheit nicht ausreichend gut umsetzbar. Innerhalb des Konsensusverfahrens sprachen sich die Expert*innen insbesondere für Mischformen aus.

### Einschlusskriterien

Im Konsensusverfahren wurde eine gemeinsame Schulung von Patient*innen mit typischen und atypischen Parkinson-Syndromen abgelehnt. Von einer separaten Schulung für Patient*innen mit atypischem Parkinson-Syndrom waren die Expert*innen jedoch überzeugt. Beides ist durch den konsentierten modularen Aufbau umsetzbar. Für die Durchführung einer separaten Schule stellt die Seltenheit der atypischen Parkinson-Syndrome allerdings ein Hindernis dar. Eine virtuelle Umsetzung der Schulung mit einer überregionalen Gruppenzusammensetzung könnte hier einen Lösungsansatz darstellen.

### Schulungskonzept und Gruppenzusammensetzung

Auch bezüglich der Gruppenzusammensetzung zeigt sich die Schwierigkeit der Entwicklung eines zu allen Anforderungen und Vorstellungen passenden Schulungskonzeptes. Die meisten der bisherigen Programme nutzten ein stadienübergreifendes Konzept, welches die Expert*innen innerhalb des Konsensusverfahrens jedoch ablehnten. Für neue Schulungskonzepte wird daher ein stadienspezifisches Konzept empfohlen.

## Limitationen

Bei der Stichprobenauswahl für das durchgeführte strukturierte Konsensusverfahren kann ein Selektionsbias zugunsten sehr engagierter Patient*innen bestehen. Auch die geringe Größe der Stichprobe und die Möglichkeit der Stichprobenverzerrung durch den Kreis der an einer digitalen Schule Teilnehmenden kann die Repräsentativität der Daten der formativen Evaluation einschränken.

## Ausblick

Die Ergebnisse dieser Studie leisten durch die Betrachtung verschiedener Blickwinkel einen wichtigen Beitrag zur Entwicklung eines evidenzbasierten Konzeptes für eine Schule, welche Menschen mit der Parkinson-Krankheit den Umgang mit ihrer Erkrankung erleichtern soll. Die hier gewonnenen Erkenntnisse können somit als Grundlage dienen, ein Schulungskonzept für eine Schule für Menschen mit der Parkinson-Krankheit in Deutschland zu implementieren. Zudem tragen sie auch zur Entwicklung von Schulungskonzepten für andere neurodegenerative Erkrankungen sowie chronische Erkrankungen des Alters bei.

## Fazit für die Praxis

- Patientenschulen unterstützen das Selbstmanagement der Patient*innen.
- Patientenschulen stellen einen zentralen Baustein für die nachhaltige Versorgung von Menschen mit chronischen neurologischen Erkrankungen dar.
- Aus der vorliegenden Arbeit können Empfehlungen für zukünftige Patientenschulen entnommen werden.
- Eine Durchführung in digitaler Form ist auch für Menschen mit der Parkinson-Krankheit umsetzbar.

### Supplementary Information

Hintergrund- und Zusatzinformationen, ergänzende Tab. 2
