# Supplementary material for: How the implementation of a school for people with Parkinson’s disease can succeed—Results of a consensus study and a formative evaluation
Source: Nervenarzt. 2024 Mar 14;95(6):539–43. [Article in German] doi: 10.1007/s00115-024-01639-z (PMC11178552; doi:10.1007/s00115-024-01639-z)
Supplement: Supplementary file 1 — Hintergrund- und Zusatzinformationen, ergänzende Tab. 2 [file 115_2024_1639_MOESM1_ESM.pdf]

Anhang zu:

Wie die Umsetzung einer Schule für Menschen mit Parkinson-Krankheit gelingen kann - Ergebnisse eines Konsensusverfahrens und einer formativen Evaluation

Tanita Gerschel[1], Scally Prokop*[1], Lara Schulze*[1], Dipl.- Psych. Tim Feige[2], Dr. rer. medic Anja Zschieschang[3], Prof. Dr. Michael Thomas Barbe[4], Robert Bitterlich[1], Julian Caffier[5], Dr. med. Ilona Csoti[6], Prof. Dr. med. Carsten Eggers[7], PD Dr. phil. Heiko Gaßner[8,9], Dr. med. Eileen Gülke[10], Dr. med. Tom Hähnel[1], Dr. med. Dipl.-Psych. Heinz Herbst[11], PD Dr. med. Angela Jochim[12], Anni Kiparski[12], PD Dr. med. Martin Klietz[13], Alexa von Liel[12], Prof. Dr. med. Paul Lingor[12], Dr. med. Kai Loewenbrück[1], Prof. Dr. med. Walter Maetzler[14], Dr. med. Dominik Pürner[12], PD Dr. med. Christoph van Riesen[15], Dr. rer. medic. Henning Schmitz-Pfeiffer[1], Dr. med. Thorsten Süß[16], Prof. Dr. med. Lars Tönges[17,18], Prof. Dr. med. Daniel Weiß[19], Prof. Dr. med. Martin Wolz[20], Prof. Dr. med. Björn Falkenburger[1]*

1: Klinik und Poliklinik für Neurologie, Universitätsklinikum Carl Gustav Carus an der Technischen Universität Dresden

2: Deutsches Zentrum für Neurodegenerative Erkrankungen (DZNE), Standort Dresden

3: Klinik und Poliklinik für Kinderchirurgie, Universitätsklinikum Carl Gustav Carus an der Technischen Universität Dresden

4: Klinik für Neurologie, Universität zu Köln, Medizinische Fakultät und Uniklinik Köln

5: Universitätsklinikum Hamburg Eppendorf

6: Fachabteilung für Neurologie, Gertrudis Klinik Biskirchen, Fachklinik für Parkinson

7: Klinik für Neurologie, Knappschaftskrankenhaus Bottrop

8: Molekulare Neurologie, Arbeitsgruppe Bewegungsanalyse und digitale Medizin, Universitätsklinikum Erlangen

9: Fraunhofer IIS, Fraunhofer Institut für integrierte Schaltungen IIS, Erlangen

10: Klinik und Poliklinik für Neurologie, Universitätsklinikum Hamburg Eppendorf

11: Neurozentrum Stuttgart

12: Klinik und Poliklinik für Neurologie, Klinikum rechts der Isar der Technischen Universität München

13: Klinik für Neurologie mit Klinischer Neurophysiologie, Medizinische Hochschule Hannover

14: Klinik für Neurologie, Universitätsklinikum Schleswig-Holstein Campus Kiel und Christian-Albrechts-Universität Kiel

15: Klinik für Neurologie, Universitätsklinikum Göttingen

16: Neurologisches Fachkrankenhaus für Bewegungsstörungen/Parkinson, Beelitz

17: Klinik für Neurologie, St. Josef-Hospital, Ruhr-Universität Bochum

18: Zentrum für Proteindiagnostik (ProDi), Ruhr-Universität Bochum

19: Klinik für Neurologie, Universitätsklinikum Tübingen

20: Klinik für Neurologie und Geriatrie, Elblandklinikum Meißen

Die Beiträge von Tanita Gerschel, Scally Prokop und Lara Schulze zu dieser Arbeit sind gleichwertig.

Material und Methoden

<u>Das strukturierte Konsensusverfahren</u>

Das strukturierte Konsensusverfahren gliederte sich in drei Phasen und wurde mit Expert*innen der Parkinson-Erkrankung durchgeführt (Abbildung 1). Als Expert*innen wurden Personen definiert, wenn sie über berufliche oder persönlich-erfahrungsbedingte Expertise verfügten. Anhand dessen konnte eine Einteilung in drei verschiedene Gruppen erfolgen: Gruppe 1, Ärztliches Personal, Psycholog*innen, Psychotherapeut*innen, Wissenschaftlich Mitarbeitende. Gruppe 2, Medizinisch-Technische Assistent*innen, Logotherapeut*innen, Ergotherapeut*innen, Physiotherapeut*innen, Case Manager*innen, Parkinson Nurses. Gruppe 3, Teilnahmefreudige Menschen mit Parkinson-Krankheit aus allen Stadien, Erfahrene Angehörige (Leiter*innen von Selbsthilfegruppen). Ein Co-Nominationsverfahren ermöglichte die deutschlandweite Rekrutierung (Abbildung 2). Dabei wurden die Ärzt*innen über die Arbeitsgruppe Netzwerke und digitale Versorgung der Deutschen Gesellschaft für Parkinson und Bewegungsstörungen (DPG) rekrutiert. Diese schlugen auch Teilnehmende der Therapeutengruppe vor. Die Expert*innen der Patientengruppe wurden über die Deutsche Parkinson Vereinigung (DPV) rekrutiert.

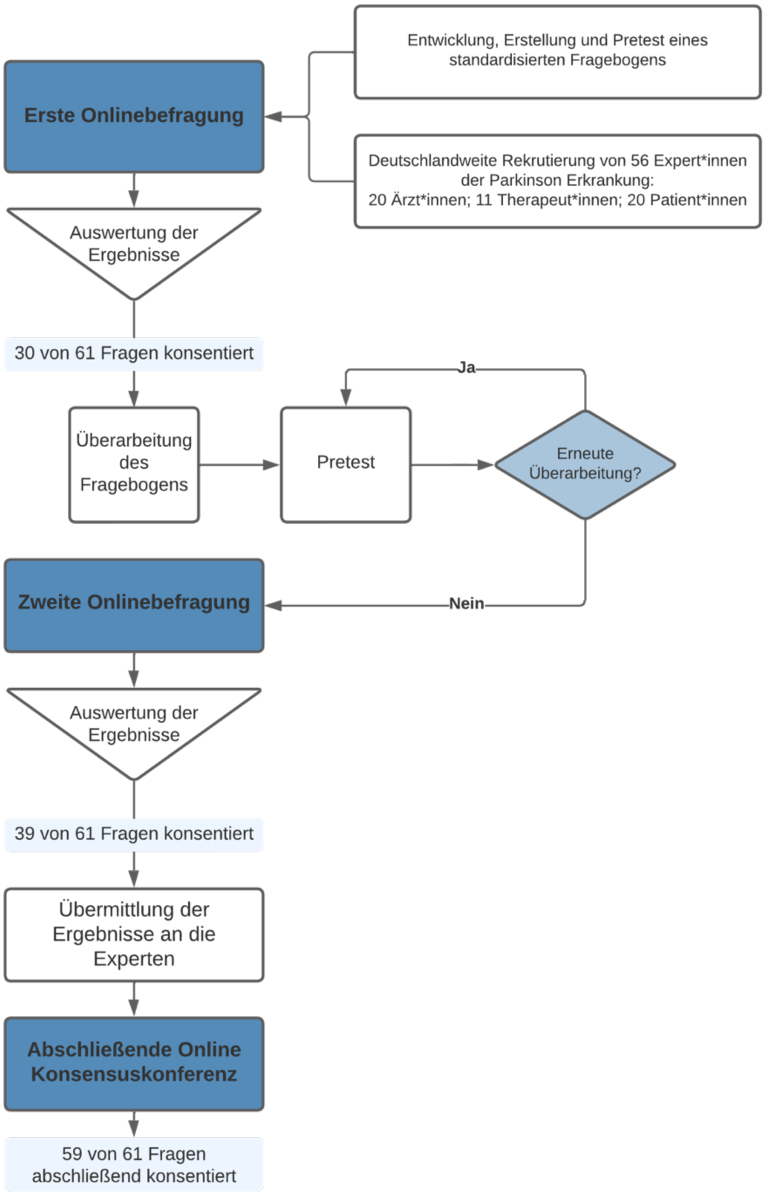

Abbildung 1: *Phasen des strukturierten Konsensusverfahrens*

Aufschlüsselung der drei Phasen des Konsensusverfahrens in einem Flussdiagramm. Die erste Phase umfasst die Entwicklung des Fragebogens sowie die erste Online-Befragung. Die zweite Phase beinhaltet die Überarbeitung des Fragebogens der ersten Phase sowie die zweite Online-Befragung. Abschließend findet eine Online-Konsensuskonferenz in der dritten Phase statt.

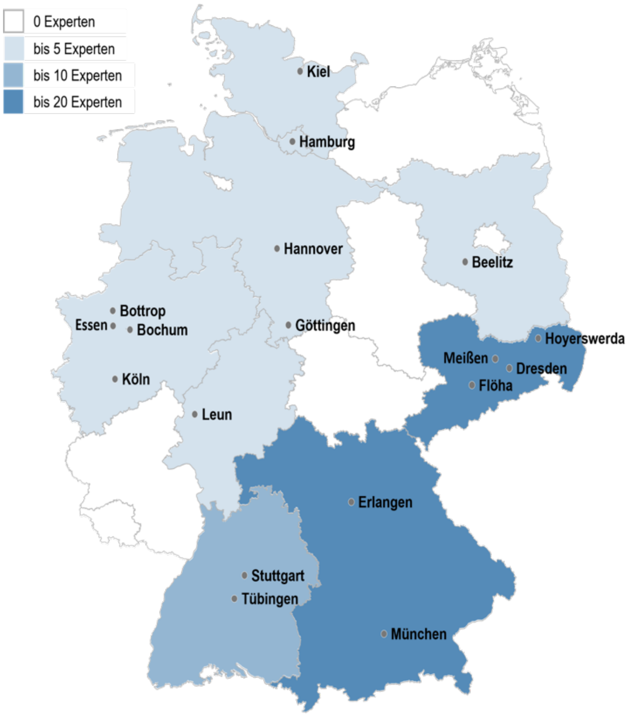

Abbildung 2: *Deutschlandweite Rekrutierung für das Konsensusverfahren*

Aufschlüsselung der rekrutierten Experten-Anzahl in den einzelnen Bundesländern von Deutschland anhand einer Karte

Die erste Phase des strukturierten Konsensusverfahrens umfasste die Entwicklung des Fragebogens sowie die erste Online-Befragung. Der entwickelte Fragebogen enthielt allgemeine Vorgaben des Zentrum Patientenschulung und Vorgaben bereits existierender Schulungsprogramme, insbesondere des Patient Education for People with Parkinson’s Disease and their Carers [10] sowie der National Parkinson School in Schweden [11]. In der zweiten Phase wurde nur über die noch nicht-konsentierten Fragen erneut abgestimmt [12], wobei Konsens bei mindestens 75 % Zustimmung und Dissens bei 75 % Ablehnung definiert wurde. In einer abschließenden Konsensuskonferenz wurde über diejenigen Fragen diskutiert und abgestimmt, zu denen nach der zweiten Befragung noch kein Konsens erreicht wurde.

Die Ergebnisse des Verfahrens wurden mit Excel deskriptiv statistisch ausgewertet. Es erfolgte zudem eine Aufschlüsselung der Ergebnisse nicht-konsentierter Fragen nach den einzelnen Expertengruppen. Qualitative Daten wie mögliche Themen und Inhalte der Patientenschule wurden mit MAXQDA (VERBI, Version: 2020.4.2) ausgewertet.

Die formative Evaluation

Die evaluierte Patientenschule fand von März bis August 2021 als Pilotprojekt in drei Gruppen statt. Die Gestaltung der Schulung orientierte sich an der schwedischen National Parkinson School und wird in Tabelle 2 näher beschrieben. Die Module begannen mit einem Rückblick auf den letzten Termin. Daran schlossen sich die erste Wissenseinheit, eine Pause und anschließend die zweite Wissenseinheit an. Zum Abschluss wurden eine Wochenaufgabe gestellt und gemeinsam Entspannungs- und Bewegungsübungen durchgeführt.

Die telefonische Rekrutierung von Patient*innen erfolgte aus einer Liste von in der neurologischen Abteilung der Uniklinik Dresden behandelten Menschen mit einer gesicherten G20-Diagnose (Stand 12/2019).

Die formative Evaluation erfolgte durch dreizehn telefonische Zufriedenheitsinterviews nach dem ersten Modul sowie insbesondere drei Fokusgruppen [13] mit insgesamt zwölf Teilnehmenden nach dem letzten Modul, wobei alle an der Patientenschule teilnehmenden Personen zur Beteiligung eingeladen waren. Die Telefoninterviews und die Fokusgruppen wurden im Programm MAXQDA mittels inhaltlich-strukturierender qualitativer Inhaltsanalyse ausgewertet [14].

Tabelle 2: *Vergleich des strukturierten Konsensusverfahrens mit der formativen Evaluation*

	**Ergebnisse des strukturierten Konsensusverfahrens (Prozent Konsens)**	**Formative Evaluation**	
		**Gestaltung der formativ evaluierten Patientenschule**	**Ergebnisse der formativen Evaluation**
**Rahmenbedingungen einer Schule für Menschen mit M. Parkinson**			
Konzept	● Regelmäßig aktualisiertes Schulungskonzept (100%)<br>● Verschriftlicht in Form eines Manuals (94,3%)<br>● Krankheitsspezifisches Konzept mit separaten Manualen für die unterschiedlichen Krankheitsstadien (93,8%)	● Manualisiertes Schulungskonzept<br>● Gemeinsame Schulung von Patient*innen in verschiedenen Krankheitsstadien	
Format	● A. e. Ambulante Durchführung (69,6%) 1)<br>● A. e. hybride Durchführung (mehrheitliche Zustimmung mit 56%) mit online und in Präsenz teilnehmenden Personen 2)<br>● Formatanpassung in reines Online-/Präsenzformat nach örtlichen Gegebenheiten, Krankheitsstadium und Patientenwunsch	● Ambulante Durchführung<br>● Reines Onlineformat über BigBlueButton	● Gutes Zurechtkommen mit BigBlueButton<br>● Positive Haltung zum digitalen Schulungsformat<br>● Wahrnehmung der Schulung im privaten Wohnumfeld als angenehm, einfach und der Konzentration zuträglich<br>● Wunsch nach persönlichem Kontakt<br>● Wunsch nach vorwiegend virtueller Umsetzung ergänzt durch Eröffnungs- und Abschlusstermin in Präsenz
Einschluss von Angehörigen	● Teilweise Teilnahme der Angehörigen an der Schule der Patient*innen (78,3%)	● Nein	● Deutlich abwehrende Haltung gegenüber einer gemeinsamen Schulung mit Angehörigen
Zeitlicher Rahmen	● Aufteilung der Schule in sechs bis acht (85,7%) wöchentlich (87%) stattfindende 60 – 90 minütige Module (85%) inklusive Kurzpause	● Sieben wöchentlich stattfindende 90 minütige Module mit einer Pause nach 45 min	● Wöchentlicher Rhythmus und Zeitumfang der Module als gut empfunden<br>● Große Bedeutung der Pause

<table>
<tr><th rowspan="2"></th><th rowspan="2">Ergebnisse des strukturierten Konsensusverfahrens</th><th colspan="2">Formative Evaluation</th></tr>
<tr><th>Gestaltung der formativ evaluierten Patientenschule</th><th>Ergebnisse der formativen Evaluation</th></tr>
<tr><td colspan="4">Qualitätskriterien einer Schule für Menschen mit M. Parkinson</td></tr>
<tr><td>Einschlusskriterien</td><td>● Möglichkeit der Teilnahme von Patient*innen mit idiopathischem Parkinsonsyndrom und atypischem Parkinsonsyndrom (78,2%)</td><td>● In der neurologischen Abteilung der Uniklinik Dresden behandelte Menschen mit einer gesicherten G20-Diagnose</td><td></td></tr>
<tr><td>Ausschlusskriterien</td><td>● Absolute Ausschlusskriterien: bedeutsame geistige Beeinträchtigungen, neuropsychiatrische Störungen, kein Verständnis der deutschen Sprache
● Relative Ausschlusskriterien für Präsenz-Format: sehr starke Behinderung und aktuell akute Erkrankung
● Relative Ausschlusskriterien für Online-Format: Blindheit</td><td></td><td></td></tr>
<tr><td>Gruppengröße</td><td>● Sechs bis acht Personen (89,1%)</td><td>● Drei, fünf und sechs regelmäßig teilnehmende Personen</td><td>● Kritik an zu geringer Personenanzahl in der Gruppe mit drei Personen, ansonsten Lob der kleinen Gruppengröße</td></tr>
<tr><td>Gruppenstruktur</td><td>● Geschlossene Gruppen, zusammengesetzt nach Kriterien (83%), wie z. B. Krankheitsstadium (75,0%)</td><td>● Geschlossene Gruppen mit Pat. unterschiedlichen Alters und Krankheitsdauer</td><td></td></tr>
<tr><td>Schulungsleitung</td><td>● Multiprofessionelles Team (96,0%) (u. A. ärztliches Personal, Parkinson Nurses und psychologisches Fachkräfte)
● Regelmäßige Schulung (100%) in Train-the-Trainer-Seminaren (94,4%)
● Voraussetzungen für Schulungsleiter: fachliche (98,2%), didaktische (89,1%), soziale (96,4%) und persönliche (89,1%) Kompetenz</td><td>● Zwei psychologische Fachkräfte</td><td></td></tr>
<tr><td>Wiederholung der Schule</td><td>● Wiederholung einzelner Module (76,0%) nach Änderung des Krankheitsstadiums (76,0%) oder auf ärztliche Empfehlung (75,0%) oder auf Patientenwunsch (79%)</td><td>● Keine</td><td>● Wunsch nach einmal jährlicher Auffrischung inklusiver neuer Inhalte</td></tr>
</table>

	Ergebnisse des strukturierten Konsensusverfahrens	Formative Evaluation	
		**Gestaltung der formativ evaluierten Patientenschule**	**Ergebnisse der formativen Evaluation**
**Umsetzungskriterien und Inhalte einer Schule für Menschen mit M. Parkinson**			
Modulbestandteile	● Modulbestandteile der National Parkinson School: Wochenaufgabe der vergangenen Woche (83,6%), Wissensvermittlung (94,5%), Gruppengespräch zum neuen Wissen (85,5%), Besprechung der neuen Wochenaufgabe (80,0%), Entspannungs-/Bewegungsübung (87,3%), Feedback (100%)	● Modulbestandteile der National Parkinson School: Vorbereitung, Wochenaufgabe der vergangenen Woche, Wissenseinheit, Wochenaufgabe, Entspannung/Bewegung, Rückmeldung	
Techniken der Wissensvermittlung	● Interaktive aktivierende Methoden (Diskussion, Kleingruppenarbeit, Gruppengespräche) und frontale Wissensvermittlung (Vortrag) (98,2%)<br>● Zusätzliche Vor- und Nachbereitungsaufgaben (84,9%)	● Frontale Wissensvermittlung<br>● Austausch der Pat. in Form von Gruppengesprächen und Diskussionen<br>● Wochenaufgaben zur Vor- und Nachbereitung	● Positive Wahrnehmung des Erfahrungsaustausches<br>● Wahrnehmung der Wochenaufgaben als überwiegend sinnvoll und in der Umsetzung von eigenen Ressourcen abhängig
Materialien	● Digitale (76,9%) und gedruckte (100%) Lernmaterialien<br>● Nutzung einer zentralen Lern- und Wissensplattform (79,6%)	● WordPress Seite als digitale Lernplattform	● Variieren der Häufigkeit der Nutzung von dreimal während der Schule bis dreimal pro Woche<br>● Wahrnehmung der Lernplattform als gut und übersichtlich mit Verbesserungspotential für einfachere Handhabbarkeit
Inhalte der Patientenschule	● Themengebiet „Was ist M. Parkinson?“ mit u. A. Symptomen, Diagnostik, Notfallsituationen und Pathophysiologie<br>● Themengebiet „Wie kann man M. Parkinson behandeln?“ mit medikamentöser, komplexer und unterstützender Therapie<br>● Themengebiet „Umgang mit M. Parkinson“ mit u. A. Selbstbeobachtung, Stressbewältigung, sozialrechtlichen Themen	● Entstehung von M. Parkinson, motorische Symptome & ihre Therapie, nicht-motorische Symptome & Umgang damit, Medikamente, Nebenwirkungen, weitere Therapieoptionen, Krankheitsverarbeitung, Selbstbeobachtung, Stressbewältigung, Kommunikation, bereichernde Aktivitäten, sozialrechtliche Themen	● Bewertung sehr positiv<br>● Beibehaltung der aktuellen Inhalte ohne Kürzung möglich<br>● Wunsch nach Aufgreifen neuester Forschungsergebnisse<br>● Anfänglich empfundene Belastung durch vermehrte Beschäftigung mit der Krankheit, aber auch positive Verstärkung und Anreize für Umsetzung eigener Ziele

1) mehrheitliche Zustimmung
2) mehrheitliche Zustimmung - weitere Aufschlüsselung: Hybridformat: 56,0%, Angebot aller Formate: 48,0%, Mischung: 36,0%, Präsenzformat: 16,0%, Videoformat: 8,0%
